# Surgical trials and trial registers: a cross-sectional study of randomized controlled trials published in journals requiring trial registration in the author instructions

**DOI:** 10.1186/1745-6215-14-407

**Published:** 2013-12-01

**Authors:** Julia LS Hardt, Maria-Inti Metzendorf, Joerg J Meerpohl

**Affiliations:** 1 Department of Surgery, University Medical Center Mannheim, University of Heidelberg, Theodor-Kutzer-Ufer 1-3, 68135 Mannheim, Germany; 2 Library, University Medical Center Mannheim, University of Heidelberg, Theodor-Kutzer-Ufer 1-3, 68135 Mannheim, Germany; 3 Department of Medical Biometry and Medical Informatics, German Cochrane Centre, University Medical Center Freiburg, Berliner Allee 29, 79110 Freiburg, Germany

**Keywords:** Trial registration, Randomized controlled trials, Surgery journals, Results reporting

## Abstract

**Background:**

Trial registration and the reporting of trial results are essential to increase transparency in clinical research. Although both have been strongly promoted in recent years, it remains unclear whether they have been successfully implemented in surgery and surgery-related disciplines. In this cross-sectional study, we assessed whether randomized controlled trials (RCTs) published in surgery journals requiring trial registration in their author instructions were indeed registered, and whether the study results of registered RCTs had been submitted to the trial register and were thus publicly available.

**Methods:**

The ten highest ranked surgery journals requiring trial registration by impact factor (Journal Citation Reports, JCR, 2011) were chosen. We then searched MEDLINE (in PubMed) for RCTs published in the selected journals between 1 June 2012 and 31 December 2012. Any trials recruiting participants before 2004 were excluded because the International Committee of Medical Journal Editors (ICMJE) first proposed trial registration in 2004. We then searched the World Health Organization (WHO) International Clinical Trials Registry Platform (ICTRP) to assess whether the identified RCTs were indeed registered and whether the results of the registered RCTs were available in the register.

**Results:**

The search retrieved 588 citations. Four hundred and sixty references were excluded in the first screening. A further 25 were excluded after full-text screening. A total of 103 RCTs were finally included. Eighty-five of these RCTs (83%) could be found via the ICTRP. For 7 of 59 (12%) RCTs, which were registered on ClinicalTrials.gov, summary study data had been posted in the results database.

**Conclusions:**

Although still not fully implemented, trial registration in surgery has gained momentum. In general, however, the submission of summary study data to ClinicalTrials.gov remains poor.

## Background

Selective reporting of study results distorts the body of evidence available for clinical decision making. In recent years, several guidelines and recommendations have been published in order to increase transparency of scientific research. One of the key policy initiatives of this development towards more transparency was the call for the obligatory registration of all clinical trials in public trial registers. In September 2004, the International Committee of Medical Journal Editors (ICMJE) proposed 'comprehensive trial registration as a solution to the problem of selective awareness’. In order to advance this goal, the ICMJE decided to require the registration in a public trials register as a mandatory condition for the consideration for the publication of a study report. The ICMJE trial registration requirement policy applies to all trials which started patient recruitment beginning 1 July 2005, and was adopted by all ICMJE member journals [1]. Since then, many other journals in addition to the ICMJE member journals have adopted the ICMJE policy.

The implementation of comprehensive mandatory trial registration would allow scientists, clinicians, and study participants to track trials and prevent biased reporting, for example the non-reporting of trials with negative or inconclusive results. Therefore, even unfavorable trial results would not be lost to the pool of medical knowledge. Based on the information provided by the register, prospective investigators would be able to formulate new research questions, plan new trials to fill the gaps in the knowledge base, and avoid unnecessary duplications [2]. Furthermore, public electronic access to all trials could inspire researchers to collaborate and could also support trial recruitment [3].

In April 2007, the ICMJE expanded the definition of clinical trials that have to be registered by adopting the World Health Organization (WHO) definition of a clinical trial, which also includes preliminary trials (phase I trials). The deadline for the implementation of these modifications was 1 July 2008 [4]. One month after the ICMJE’s expansion of the definition, in May 2007, the WHO launched its International Clinical Trials Register Platform (ICTRP) in order to offer an international portal for identifying, deduplicating, and searching trials from registers all over the world. The ICTRP requires a minimum trial registration data set consisting of 20 items [2], which is also supported by the ICMJE [5]. As of 1 July 2007, the member journals of the Surgery Journal Editors Group (SJEG) require registration of all prospective clinical trials prior to the enrollment of the first patient [6]. Trials which had started recruitment before the deadline had to register before editorial review. Manuscripts are now required to specify the registration number in the abstract [7].

But how did all these policy recommendations, regulations, and statements influence the practice of trial registration? By examining the development and growth of ClinicalTrials.gov, the largest public trial register, which was created as a result of the Food and Drug Administration Modernization Act (FDAMA) in 1997, it can be concluded that trial registration has gained momentum and that there has been major progress within the last decade. As of 13 November 2013, ClinicalTrials.gov included more than 154,000 studies from across all 50 American states and 185 countries worldwide (http://www.clinicaltrials.gov/ct2/resources/trends).

In September 2008, the ClinicalTrials.gov results database was launched to meet the requirement in Section 801 of the Food and Drug Administration Amendments Act (FDAAA 801) that study sponsors or principal investigators report basic results for 'applicable clinical trials’ (ACTs). A trial is considered 'applicable’ if it meets the following criteria: phase II to IV interventional study involving drugs or medical devices regulated by the FDA; at least one site in the USA; and initiated or ongoing as of 27 September 2007, or later [8]. For all applicable trials, the results have to be submitted no later than 12 months after the trial’s completion date (http://www.clinicaltrials.gov/ct2/manage-recs/fdaaa - WhenDoINeedToRegister). The summary results data in the results database are presented mainly in a tabular format and are publicly accessible. This does not only benefit researchers and journal editors but also patients and the general public. The main objectives of the ClinicalTrials.gov results database are to reduce publication bias and selective outcome reporting and to promote complete reporting by structured data entry [9].

Although the facts and numbers presented above indicate a remarkable success of the initiatives and efforts to promote trial registration and results reporting, it remains unclear whether these have been successfully implemented as integral parts of clinical research in surgery and surgery-related disciplines. We therefore chose to explore whether randomized controlled trials (RCTs) published in the ten highest ranked (by impact factor) surgery journals that require trial registration in their author instructions were indeed registered. We also chose to address the question of whether the study results of the registered RCTs were publicly available on the trial register website.

## Methods

We accessed the Journal Citation Reports (JCR) Science Edition 2011 on 8 January 2013. Two authors (JH and MIM) independently identified the first ten journals among the top surgery journals by impact factor that required trial registration in the author instructions on their websites (Table 1). All journals not explicitly requiring trial registration in their author instructions were excluded. We intentionally chose the ten journals with the highest impact factors assuming that their policies and publishing practices would meet the current highest standards. Moreover, we expected that trialists publishing in such top-class journals would be more likely to act in an exemplary manner with regard to trial registration and results reporting.

**Table 1:** The first ten journals among the top surgery journals by impact factor that required trial registration in the author instructions

**Journal**	**Country**	**Impact factor (JCR 2011)**
*Annals of Surgery*	USA	7.492
*American Journal of Transplantation*	USA	6.394
*Endoscopy*	Germany	5.210
*Journal of Neurology, Neurosurgery & Psychiatry*	UK	4.764
*British Journal of Surgery*	UK	4.606
*Journal of the American College of Surgeons*	USA	4.549
*Archives of Surgery* (*JAMA Surgery* since 1 January 2013)	USA	4.422
*Surgical Endoscopy*	Germany	4.013
*Transplantation*	USA	4.003
*Surgery for Obesity and Related Diseases*	USA	3.929

The *American Journal of Surgical Pathology* (impact factor 4.352) and *Annals of Surgical Oncology* (impact factor 4.166) were excluded because the author instructions of these journals did not require trial registration. JCR, Journal Citation Reports.

In a second step, MEDLINE was searched via PubMed for RCTs published in these journals between 1 June 2012 and 31 December 2012. All of the included journals are fully indexed in the MEDLINE database. For the identification of RCTs in MEDLINE (search conducted 15 February 2013), we applied the sensitivity-maximizing Cochrane Highly Sensitive Search Strategy for identifying RCTs in the PubMed format [10]. The search strategy was slightly modified to fit the surgical setting (Additional file 1).

One author (JH) screened titles and abstracts, excluded clearly irrelevant references, and downloaded full-texts of all potentially relevant citations. Then, two authors (JH and MIM) independently screened the full-texts and excluded non-randomized studies. Trials recruiting participants prior to 2004 were also excluded because the ICMJE first proposed comprehensive trial registration in a statement published in September 2004, which requires registration in a public trials register for trials that started enrollment after 1 July 2005 only. All discrepancies were resolved by re-examining the full-texts and in discussion with a third author (JM).

In the next step, two authors (JH and MIM) independently searched the ICTRP (**27** February 2013) for information on the registration of all included RCTs. Trials were searched for either with the registration identification quoted in the publication or, if this identification was not provided, with different keywords describing the topic of the trial. We searched using the ICTRP’s standard search form, which searches within the title, primary sponsor, health condition(s), intervention(s), countries of recruitment, main identification, and secondary identification(s) of the trial data. Additionally, we used Boolean operators to broaden or narrow the search. If such a search was not successful, we took a more sensitive approach by searching first for the main author’s name, second for the most specific terms of the institutional name stated in the authors’ affiliations, or third for the country or city the trial was conducted in combined with one or two specific terms describing the trial. Trials not found through these extensive ICTRP searches were considered to be unregistered.

Reviewing the full-texts and the information given on the trials register website, we collected data regarding the following topics and parameters: sample size, country of main investigator, national versus multinational and monocenter versus multicenter setting, surgical subspecialty, study objective, and start of patient recruitment. Furthermore, we extracted information on whether trial registration was explicitly mentioned in the article, meaning whether the article included at least one full sentence describing that the trial was registered in a specific trial register. We also reviewed whether the registration number was specified in the title, abstract, or main text, since the SJEG member journals as well as the ICMJE demand specifications of the trial registration number in the abstract as evidence of registration [7]. Moreover, the ICMJE even recommends that authors list the trial registration number the first time a trial acronym is used in the manuscript (http://www.icmje.org/publishing_10register.html).

Finally, the primary trial registers were checked for study results of the registered RCTs. We defined study results as either a citation to a publication reporting the trial or, in the case that ClinicalTrials.gov was the primary register, if aggregate summary data were provided in addition to a citation. Two authors (JH and MIM) extracted the following information for the registered RCTs: primary register; link to a PubMed citation or list of publication(s) provided by the investigators; and, if ClinicalTrials.gov was the primary register: aggregate summary study data posted in the ClinicalTrials.gov results database; automatic link to a PubMed citation mapped via the ClinicalTrials.gov identifier (NCT number); and study start date and study registration date ('study first received’ in ClinicalTrials.gov) in order to identify whether trials were registered retrospectively or prospectively.

We did not explicitly assess if the RCTs registered in ClinicalTrials.gov were ACTs according to the FDAAA 801. Ethics approval was not required for this study.

## Results

There were 199 journals in the subject category 'surgery’ indexed in JCR 2011. The first ten surgery journals with the highest impact factors ranging from 7.492 to 3.929, which explicitly required trial registration in their online author instructions, were chosen (Table 1).

**Figure 1:**
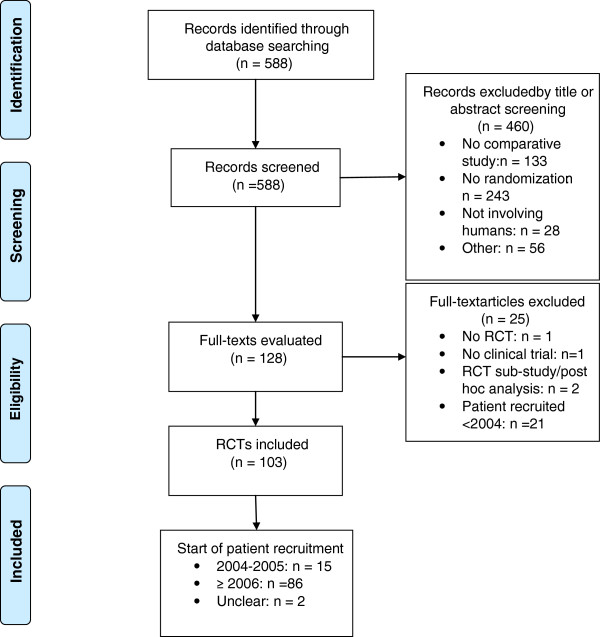
**Study flow diagram: selection process of RCTs.** RCT, randomized controlled trial.

The search for RCTs published between 1 June 2012 and 31 December 2012 retrieved 588 citations. From these, 460 clearly irrelevant references were excluded by title or abstract screening. We then evaluated the full-texts of the remaining 128 references (Additional file 2) and excluded 25 of these for the following reasons: 21 studies had started patient recruitment prior to 2004, two citations reported sub-studies of older RCTs, one reported a non-randomized trial, and one was a study not concordant with the WHO definition of a clinical trial (http://www.who.int/ictrp/en/). This last study had investigated whether there were any differences in the learning outcomes of healthy participants who had trained to proficiency on low- or high-fidelity laparoscopic surgical simulators. The remaining 103 citations were further investigated. The majority of the RCTs (n = 86; 83%) had initiated patient recruitment during or after 2006, 15 RCTs (15%) had started to recruit participants in 2004 or 2005, and two RCTs (2%) did not state the start of recruitment (Figure 1).

## Trial registration

Eighty-five of the 103 analyzed RCTs (83%) could be identified in the ICTRP (Table 2; Additional file 3). Of these 85 RCTs, 15 (18%) had been registered prospectively, 45 (53%) had been registered retrospectively, and 21 (25%) had been registered within the same month as the study start date. For the remaining four studies, we were not able to find information on the study start and registration dates for the following reasons: three of the RCTs were only registered in the European Union Clinical Trials Register (EU-CTR), which does not provide the date of study registration, and one RCT was registered in a Belgian register, which is publicly not accessible. All RCTs that were prospectively registered had enrolled the first patient in or after 2006. None of the RCTs which started patient recruitment in 2004 or 2005 had been registered prospectively (Table 2).

**Table 2:** Registration of RCTs stratified by start of patient recruitment

	**Start of patient recruitment**			
	**All**	**2004 to 2005**	**During or after 2006**	**Unclear**
	**n = 103 (100%)**	**n = 15 (100%)**	**n = 86 (100%)**	**n = 2 (100%)**
Registered RCTs	85 (83%)	14 (93%)	71 (83%)	0
Registration (number) mentioned in article	68 (80.0%)	11 (71.4%)	57 (70.4%)	
Registration (number) not mentioned in article	17 (20.0%)	3 (21.4%)	14 (19.7%)	
Registered prospectively	15 (17.6%)	0	15 (21.1%)	
Registered retrospectively	45 (52.9%)	12 (85.7%)	33 (46.5%)	
Study start and registration within same month	21 (24.7%)	1 (7.1%)	20 (28.2%)	
Unclear time relation between study start and registration	4 (4.7%)	1 (7.1%)	3 (4.2%)	
Not registered	18 (17%)	1 (7%)	15 (17%)	2 (100%)

RCT, randomized controlled trial.

Seventeen (81%) of the 21 excluded RCTs with patient recruitment before 2004 had been registered. All of them had been retrospectively registered after enrollment of the first patient.

Sixty-eight (80%) of the 85 registered trials specified the registration identifier: 25 in the main text only, 21 in the abstract and main text, ten in the abstract only, two in the title and main text, one in the acknowledgments section, and nine below or above the list of author affiliations (two of them additionally specified the identifier in the main text and abstract, respectively). The 17 (20%) registered trials without specification of the registration number were not classified as 'registered’ unless they were found in the ICTRP searching with words extracted from the publication. One RCT which specified the registration number in the abstract and main text reported the wrong number twice. The reported number actually belonged to another RCT of the same first author, and we discovered the correct NCT identifier by searching the ICTRP. Moreover, we found four registered RCTs which were categorized as a prospective cohort study (n = 3) or prospective case–control study (n = 1).

Forty-four of the 68 (65%) RCTs specifying the registration number also mentioned trial registration explicitly in a full sentence in the abstract and/or main text.

Eighteen of the 103 included RCTs (17%) did neither report trial registration nor could they be found in the ICTRP. Upon examination of the unregistered RCTs, we noted the following differences to the registered RCTs: all these RCTs were national and all had been undertaken in a single-center setting, except for three RCTs conducted at two to three centers. In addition, it seemed that the median sample size was smaller (66 versus 126; Table 3).

**Table 3:** Study characteristics of the included RCTs

**Study characteristic**	**Registered RCTs (n = 85)**	**Unregistered RCTs (n = 18)**
National, multinational setting	68 (80%), 17 (20%)	18 (100%), 0
Monocenter, multicenter setting	49 (58%), 36 (42%)	15 (83%), 3 (17%)
Sample size (median (range))	126 (12 to 66,000)	66 (12 to 256)
Country of main investigator	USA (n = 13), Germany (n = 8), Netherlands (n = 7), China (n = 6), Italy (n = 6), Norway (n = 6), UK (n = 5), Japan (n = 4), Austria (n = 3), Belgium (n = 3), Denmark (n = 3), South Korea (n = 3), Spain (n = 3), Sweden (n = 3), Switzerland (n = 3), Finland (n = 2), Egypt (n = 2), New Zealand (n = 2), and Australia, France, and India (for all n = 1)	South Korea (n = 5), USA (n = 5), Brazil (n = 2), UK (n = 2), and China, Finland, India, and Ukraine (for all n = 1)
Surgical subspecialty or surgery-related discipline	General surgery (n = 24), endoscopy/gastroenterology/gastrointestinal surgery (n = 14), nephrology/kidney transplantation/surgery (n = 10), bariatric surgery (n = 6), hepatology/liver transplantation/hepatobiliary surgery (n = 6), anesthesiology/surgery (n = 5), pediatrics/pediatric surgery (n = 3), psychiatry/neurology/surgery (n = 5), cardiology/heart transplantation/cardiothoracic surgery (n = 2), colorectal surgery (n = 2), and other (n = 8)	General surgery (n = 9), gastroenterology/gastrointestinal endoscopy/gastrointestinal surgery (n = 3), anesthesiology (n = 2), neurosciences/neurology/psychiatry/otolaryngology (n = 2), pediatric surgery (n = 1), and reconstructive breast surgery (n = 1)

RCT, randomized control trial.

### Reporting results

Sixty-one of the 85 registered RCTs were registered on ClinicalTrials.gov. One of these RCTs was still ongoing and published as a study protocol only, another one had been withdrawn before enrollment of the first patient. Both were excluded from further analysis, and the remaining 59 RCTs were included. For 7 (12%) of them, results had been posted in the results database (Table 4). In our sample, the proportion of RCTs with summary data posted on ClinicalTrials.gov was smaller among the retrospectively registered trials in comparison to RCTs with prospective registration (3/45 (7%) versus 3/15 (20%); relative risk 0.33; 95% confidence interval 0.08, 1.48; *P* = 0.15).

**Table 4:** Results availability for registered RCTs stratified by completion date

	**Study completion date**				
	**All**	**Before or during February 2011**	**After February 2011 to February 2012**	**After February 2012**	**Unclear**
	**n = 59 (100%)**	**n = 24 (100%)**	**n = 25 (100%)**	**n = 5 (100%)**	**n = 5 (100%)**
**Results posted**	7 (12%)	5 (21%)	2 (8%)	0	0
Results posted only	2 (3%)	1 (4%)	1 (4%)	0	0
Results posted and link to publication provided by investigator	1 (2%)	0	1 (4%)	0	0
Results posted and automatic linkage via register identification	4 (7%)	4 (17%)	0	0	0
**No results posted**	52 (88%)	19 (79%)	23 (92%)	5 (100%)	5 (100%)
Link to publication provided by investigator only	1 (2%)	0	1 (4%)	0	0
Link to publication provided by investigator and automatic linkage via register identification	1 (2%)	0	1 (4%)	0	0
Automatic linkage via register identification only	27 (46%)	9 (38%)	15 (60%)	1 (20%)	2 (40%)
No results posted and no link provided	23 (39%)	10 (42%)	6 (24%)	4 (80%)	3 (60%)

List of registries included in the ICTRP search portal (September 2013): Australian New Zealand Clinical Trials Registry (ANZCTR); ClinicalTrials.gov; European Union Clinical Trials Register (EU-CTR); International Standard Randomised Controlled Trial Number Register (ISRCTN); Brazilian Clinical Trials Registry (ReBec); Chinese Clinical Trial Registry (ChiCTR); Clinical Trials Registry - India (CTRI); Clinical Research Information Service (CRiS), Republic of Korea; Cuban Public Registry of Clinical Trials (RPCEC); German Clinical Trials Register (DRKS); Iranian Registry of Clinical Trials (IRCT); Japan Primary Registries Network (JPRN); Pan African Clinical Trial Registry (PACTR); Sri Lanka Clinical Trials Registry (SLCTR); and The Netherlands National Trial Register (NTR). ICTRP, International Clinical Trials Register Platform; RCT, randomized controlled trial.

As mentioned in the Methods section, we did not explicitly assess whether the RCTs registered in ClinicalTrials.gov were ACTs according to the FDAAA 801. However, there are several reasons to assume that most of the included RCTs were not ACTs. First, not all of them had at least one site in the USA and were initiated or ongoing as of 27 September 2007, or later. Second, several included RCTs compared surgical procedures instead of drug or device interventions. Thus, we presume that most of the analyzed trials were not required to report summary data to ClinicalTrials.gov according to the FDAAA 801.

Twenty-five RCTs had been registered in trial registers other than ClinicalTrials.gov: International Standard Randomised Controlled Trial Number Register (ISRCTN; n = 7), EU-CTR (n = 4), The Netherlands National Trial Register (NTR; n = 3), Australian New Zealand Clinical Trials Registry (ANZCTR; n = 2), German Clinical Trials Register (DRKS; n = 2), Japan Primary Registries Network University Hospital Medical Information Network (JPRN-UMIN; n = 4), Belgian register (n = 1), Chinese Clinical Trial Registry (ChiCTR; n = 1), and Clinical Trials Registry - India (CTRI; n = 1). Of these registers, DRKS, ISRCTN, CTRI, ANZCTR, NTR, and JPRN-UMIN provide a data field for a link to publications on the study record page. The study record page of only 5 (26%) of the 19 RCTs primarily registered in these registers included a link to PubMed citations or a list of publications. EU-CTR and ChiCTR did not provide a data field for links to publications, and the Belgian register is not publicly accessible.

## Discussion

Eighty-five of the 103 analyzed RCTs (83%) were registered and 80% (68/85) of the registered trials specified the registration identifier, for example the NCT number, in the study report. In addition, 65% (44/68) of RCTs specifying the registration number also mentioned trial registration explicitly in a full sentence in the abstract and/or main text. Though the ICMJE trial registration policy requires trial registration before the enrollment of the first patient, only 18% of the registered trials had been registered prospectively. The majority were registered retrospectively (53%) or within the same month as the study start date (25%). This implies that with regard to the vast majority of registered trials, it cannot be excluded that initial details of study design, objective, eligibility criteria, or primary and secondary outcomes were changed after study start. For this specific reason, retrospective registration is only suboptimal. On the other hand, retrospective registration is helpful for the identification of trials, especially those still ongoing or not yet published.

The results of only 7 (12%) of the 59 RCTs registered on ClinicalTrials.gov had been submitted to the ClinicalTrials.gov results database. As mentioned before, we did not assess whether these 59 RCTs were ACTs. Thus, it remains unclear whether the legal requirements to submit aggregate summary data to ClinicalTrials.gov really pertain to the included RCTs. Nonetheless, investigators of all trials registered in ClinicalTrials.gov can voluntarily submit summary data to the results database. Though the legal requirement to report results applies only to certain interventional trials, sponsors and investigators should be encouraged to use the results database for timely dissemination of their research findings publicly [11].

For 28 (47%) of the trials registered on ClinicalTrials.gov there was at least a link provided to publications, which are automatically mapped to these studies by the ClinicalTrials.gov identifier (NCT number). In 29 (48%) cases there was no reporting of results or link to publications available at all.

Nguyen *et al*. recently published data on the public availability of trial results assessing cancer drugs in the USA. They analyzed 646 trials (including 209 RCTs) regarding results posting at ClinicalTrials.gov and/or publication of results in journals. One year after the completion of the trials, the results of only 9% of all trials (12% of the RCTs) were available at ClinicalTrials.gov [12]. These data are similar to our own results. Moreover, Nguyen *et al*. reports that, despite the FDAAA, results of almost half of the trials assessing cancer drugs were not publicly available (neither at ClinicalTrials.gov nor in journals) three years after completion of the trials [12].

Jones *et al*. recently conducted a cross-sectional analysis of 585 trials with at least 500 participants, which were prospectively registered with ClinicalTrials.gov to estimate the frequency with which results of large RCTs registered with ClinicalTrials.gov are not publicly available. Almost one third (n = 171) of the included 585 RCTs remained unpublished. Of the 171 unpublished RCTs, almost 80% (n = 133) had no results available in the ClinicalTrials.gov results database [13].

Comparing our findings regarding trial registration to those from disciplines not related to surgery, it seems that the awareness of the need for trial registration has grown in the surgical research community. The proportion of registered RCTs in this study by far exceeds the reported numbers from other recent trials. Milette *et al*. investigated the transparency of outcome reporting and trial registration of RCTs published in top psychosomatic and behavioral health journals between January 2008 and September 2009 [14]. Of the 63 articles reviewed, only 13 (20.6%) had been registered. A similar proportion of registered trials were reported by McGee *et al*. who conducted a cohort study of all RCTs in kidney transplantation published between October 2005 and December 2010 and determined trial registration and declaration of registration by authors [15]. Of the 307 included trials, only 74 (24%) had been registered; 44 (59%) of the registered trials declared trial registration details at least within one study report. Moreover, the authors investigated factors associated with trial registration. Trial registration was more likely if the trial was published more than once, in later years, or if it was reported in journals following the ICMJE guidelines. Furthermore, trials conducted in the USA were significantly more likely to be registered than European trials. Trial registration was also less likely for trials not declaring their funding source. Regarding the factors associated with declaration of registration details, McGee *et al*. found that registered trials were more likely to declare registration details in related reports if they were published in a journal complying with the ICMJE guidelines or in later years (2007 to 2010). Compared to European trials, trials conducted globally were less likely to declare registration details. Interestingly, USA trials were no more or even less likely to declare registration details than trials conducted in Europe.

Califf *et al*. recently examined the characteristics of clinical trials (in three different medical specialties: cardiovascular, mental health, oncology) registered in ClinicalTrials.gov. Their analysis showed that the proportion of prospectively registered trials increased over time (from 33% in October 2004 to September 2007 to 48% in October 2007 to September 2010) [16]. This is concordant with our results (Table 2).

Reveiz *et al*. investigated another important aspect of trial registration: its potential influence on reporting quality [17]. The authors conducted a cross-sectional study of 148 RCTs from the highest ranked journals (JCR 2006) and analyzed this sample with regard to adherence to key methodological items of the Consolidated Standards of Reporting Trials (CONSORT) statement and several other secondary outcomes, *inter alia* trial registration. Of these, 36% of the included RCTs reported trial registration. Reporting quality was significantly better if trial registration was declared in the trial report.

Several studies have examined whether journals publishing original articles in specialties such as urology and pediatrics endorse recommendations aimed at the improvement of publication practice. Meerpohl *et al*. analyzed the online author instructions of 69 journals indexed in the subject category 'pediatrics’ of JCR 2007 with regard to endorsement of the Uniform Requirements for Manuscripts (URM) of the ICMJE, of five major reporting guidelines, disclosure of conflicts, and trial registration [18]. Only 16 (23%) of the included 69 journals either recommended or required trial registration. This means that more than three quarters of pediatric journals did not require/recommend trial registration in August 2008. One year later, Meerpohl *et al*. analyzed 41 pediatric open access journals with regard to good publication practice [19]. The authors came to the conclusion that pediatric open access journals mention certain recommendations and guidelines, for example the URM, more frequently than conventional journals, but that the endorsement was still only moderate. Trial registration, for example, was only recommended/required by approximately a third (32%) of the included journals.

Kunath *et al*. conducted a cross-sectional study of RCTs published in 2009 in urology-related journals indexed in JCR 2009 [20]. Of the 106 included RCTs, 63 (59.4%) were registered. The proportion of reports of registered trials was significantly higher in journals requiring trial registration as a requirement for publication than in journals not mentioning trial registration in their author instructions (71.4% versus 51.6%).

It is, however, not the journal editors’ main responsibility to ensure good publication practice, especially complete trial registration. Primarily, trialists are in charge of registering their trials. Reveiz *et al*. surveyed the corresponding authors of a random sample of 500 clinical trials published between May 2005 and May 2006 [21]. Of the 275 trialists who completed the questionnaire, 64% supported the registration of all 20 items of the WHO minimum data set that should be recorded for clinical trial registration, while 6% did not support any of them. Only 21% of the respondents had registered all of their trials since 2005. However, 47% declared the intention to provide all 20 items of the WHO data set to a publicly accessible register for future clinical trials. Comparing the respondents who received mixed or only industry funding with those receiving only non-industry funding, the latter were significantly more likely to intend to provide all 20 WHO data set items for future trials.

Looking into the future of trial registration and reporting, their successful implementation as integral parts of clinical research highly depends on the continuous efforts and initiatives taken by trialists, journal editors, ethic boards, and funders.

There are some limitations to this study. We studied a cohort of RCTs published between June 2012 and December 2012. Due to the moderate sample size, the generalizability of the results might be limited. RCTs could have been missed, because the PubMed search was performed only 6 weeks after the evaluated time period. Some citations might not yet have been fully indexed with MeSH terms in MEDLINE. However, the Cochrane RCT filter does not only use MeSH terms to identify RCTs, but also text words within the database’s title/abstract field, which have been validated for identifying RCTs. Thus, the chance to have missed publications reporting an RCT not yet indexed with MeSH terms is relatively low. It is also possible that we erroneously declared a trial as unregistered if it was registered within a register not included in the ICTRP search platform and the registration was not mentioned in the publication. In addition, since the analyzed cohort of RCTs was taken from the ten journals with the highest impact factors (according to JCR 2011) which explicitly required trial registration in their instructions to authors, our results might overestimate the compliancy with trial registration and therefore might not be transferable to the entirety of surgery-related journals. This likely implies a limited external validity of our results.

## Conclusions

Although still suboptimal, the situation is improving over time and trial registration is gaining momentum. However, complete prospective trial registration has not yet been achieved even in top surgery journals, which explicitly require trial registration in their author instructions. Furthermore, the results reporting process, for example the submission of study results to the ClinicalTrials.gov results database, is still not widely practiced. Researchers, peer reviewers, and journal editors should therefore continue to collaborate to improve trial reporting and registration.

## Abbreviations

ACT: Applicable clinical trial; ANZCTR: Australian New Zealand Clinical Trials Registry; ChiCTR: Chinese Clinical Trial Registry; CONSORT: Consolidated Standards of Reporting Trials; CTRI: Clinical Trials Registry - India; DRKS: German Clinical Trials Register; EU-CTR: European Union Clinical Trials Register; FDAAA: Food and Drug Administration Amendments Act; FDAMA: Food and Drug Administration Modernization Act; ICMJE: International Committee of Medical Journal Editors; ICTRP: International Clinical Trials Register Platform; ISRCTN: International Standard Randomised Controlled Trial Number; JCR: Journal Citation Reports; JPRN: Japan Primary Registries Network; NTR: The Netherlands National Trial Register; RCT: Randomized controlled trial; SJEG: Surgery Journal Editors Group; UMIN: University Hospital Medical Information Network; URM: Uniform Requirements for Manuscripts; WHO: World Health Organization.

## Competing interests

The authors declare that they have no competing interests.

## Authors’ contributions

JH conceived and designed the study, undertook data extraction, analysis, and interpretation, and drafted and revised the manuscript. MIM designed and executed the search strategies, undertook search documentation, data extraction, analysis, and interpretation, and revised the manuscript. JM conceived and designed the study, undertook data analysis and interpretation, and drafted and revised the manuscript. All authors read and approved the final manuscript.

## Supplementary Material

Additional file 1 PubMed search strategy for identifying RCTs in 10 surgery journals. Click here for file

Additional file 2 Extracted data of 128 references evaluated as full texts. Click here for file

Additional file 3 Extracted data of 85 registered RCTs. Click here for file
