# Supplementary material for: Surgical trials and trial registers: a cross-sectional study of randomized controlled trials published in journals requiring trial registration in the author instructions
Source: Trials. 2013 Dec 1;14:407. doi: 10.1186/1745-6215-14-407 (PMC4220812; doi:10.1186/1745-6215-14-407)
Supplement: Additional file 1 — PubMed search strategy for identifying RCTs in 10 surgery journals. [file 1745-6215-14-407-S1.docx]

| **Search strategy for identifying randomized controlled trials published in the 10 surgery journals with the highest impact factor (2011) requiring trial registration in their author instructions** |
| --- |
| Database: PubMed (includes “MEDLINE”, “PubMed - in process”, “PubMed - as supplied by publisher” and “PubMed” records)  Searched: 15/02/2013  Responsible searcher: Metzendorf |
| 1. "Annals of surgery"[Journal]  2. "American journal of transplantation : official journal of the American Society  of Transplantation and the American Society of Transplant Surgeons"[Journal]  3. "Endoscopy"[Journal]  4. "Journal of neurology, neurosurgery, and psychiatry"[Journal]  5. "The British journal of surgery"[Journal]  6. "Journal of the American College of Surgeons"[Journal]  7. "Archives of surgery (Chicago, Ill. : 1960)"[Journal]  8. "Surgical endoscopy"[Journal]  9. "Transplantation"[Journal]  10. "Surgery for obesity and related diseases : official journal of the American  Society for Bariatric Surgery"[Journal])  11. 1. OR 2. OR 3. OR 4. OR 5. OR 6. OR 7. OR 8. OR 9. OR 10.  12. randomized controlled trial[pt]  13. controlled clinical trial[pt]  14. randomized[tiab]  15. randomly[tiab]  16. trial[tiab]  17. groups[tiab]  18. 12. OR 13. OR 14. OR 15. OR 16. OR 17.  19. 11. AND 18.  20. "2012/06/01"[pdat] : "2012/12/31"[pdat]  21. 19. AND 20.  22. "animals"[mh] NOT "humans"[mh]  23. 21 NOT 22 |
